# Performance of a supervised nurse-led outpatient clinic for difficult-to-manage gout patients at a tertiary center

**DOI:** 10.1186/s41927-026-00662-8

**Published:** 2026-06-03

**Authors:** Piroska Maurer, Regina Herren, Tobias Manigold

**Affiliations:** 1 Faculty of Medicine, University of Bern, Bern, Switzerland; 2 Department of Rheumatology and Immunology, Inselspital, Bern University Hospital, Bern, Switzerland; 3 Department of Rheumatology and Rehabilitation, Kantonsspital Baden AG, Im Ergel 1, Baden, 5404 Switzerland

**Keywords:** Gout, Nurse-led management, Difficult-to-manage gout, Tertiary care, Transplant patients

## Abstract

**Background:**

Gout is among the most prevalent arthritides worldwide and is associated with high morbidity, cardiovascular risk, and substantial healthcare burden. Despite effective urate-lowering therapies (ULT), many patients remain undertreated, particularly those with severe or complex disease. Nurse-led gout management has improved outcomes in primary care, but data in multimorbid tertiary center populations are lacking. We aimed to evaluate the effectiveness of a supervised nurse-led gout clinic in achieving and maintaining serum urate (SU) targets in a tertiary hospital cohort.

**Methods:**

In this observational study at the University Hospital (Inselspital) Bern we report on the performance during May 2023 and Jan 2026 in 69 patients with confirmed gout per a restricted panel of EULAR/ACR 2015 classification criteria. Patients were assigned to SU targets of ≤ 300 µmol/L (group 1: severe gout) or ≤ 360 µmol/L (group 2: non-severe gout). An Advanced Practice Nurse (APN) provided education, a rheumatologist provided pharmacological management per 2016 EULAR/ACR guidelines. SU levels, flare frequency, prophylaxis, and treatment adjustments were monitored, with follow-up at six and twelve months.

**Results:**

Group 1 required higher allopurinol doses than group 2 (*p* ≤ 0.01). SU targets were achieved in 80% of group 1 regimens and 68% of Group 2. Flare rates and prophylaxis use were comparable. At six/twelve months follow-up, 62/67% of group 1 and 85/88% of group 2 maintained SU targets. Failures were often due to external treatment modifications. No major ULT-related adverse events were observed.

**Conclusion:**

Supervised nurse-led gout management effectively achieves SU targets in this difficult-to-manage cohort. Maintaining SU targets may benefit from shorter follow-up intervals and enhanced education for patients and healthcare providers.

## Introduction

Gout is among the most prevalent arthritides worldwide, and its incidence continues to rise due to population growth and aging [5]. Gouty arthritis causes substantial patient morbidity, including immobility and frequent emergency visits, and carries both direct medical costs and indirect societal costs, such as work absenteeism [21]. Beyond the socio-economic impact, gout is an independent cardiovascular risk factor, associated with increased morbidity and mortality [3, 7]. Despite being a treatable disease, many patients remain undertreated. Surveys indicate that only 43% of general practitioners correctly identify the target serum urate (SU) levels, and just 32% advocate long-term urate-lowering therapy (ULT) [1]. Rheumatologists are heavily involved when gout becomes symptomatic; however, studies show that only 56% of patients attended by rheumatologists had at least one SU measurement [9]. In an UK national audit, although rheumatologists initiated or adapted ULT in 76% of indicated patients, only 45% achieved SU ≤ 368 µmol/L at 12 months [19], highlightening challenges in achieving optimal control even in specialty care.

Nurse-led management of gout has been shown to outperform usual care in primary care settings, improving SU target attainment, adherence, patient education, satisfaction, flare frequency, and cost efficiency [6, 8, 12, 17, 24]. Whether a similar model would work in tertiary centers managing patients with complex comorbidities and multiple therapeutic restrictions is unclear. These patients often require involvement of multiple disciplines and are more likely to meet descriptions for difficult-to-manage gout [15, 20], though no validated definition criteria exist. Building on encouraging results from nurse-led outpatient studies [6, 17], we implemented a supervised gout outpatient clinic at the University Hospital Bern in 2023 and herein report our experiences in 69 gout patients, focusing on treatment efficacy and follow-up at six and twelve months.

## Methods

### Study design

This observational study was conducted at the University Hospital Bern, Switzerland, between May 2023 and January 2026. All patients gave informed consent in accordance with the Declaration of Helsinki and following the Swiss Humanforschungsgesetz. The study was approved by the Cantonal Ethics Committee Bern (No. 2025 − 01880). Admissions from internal departments (internal medicine, emergency medicine, cardiology, hepatology, nephrology, and rheumatology) as well as external general practitioners and subspecialities for evaluation of confirmed or suspected gout were considered for inclusion in the study.

### Patient selection

**Fig. 1:**
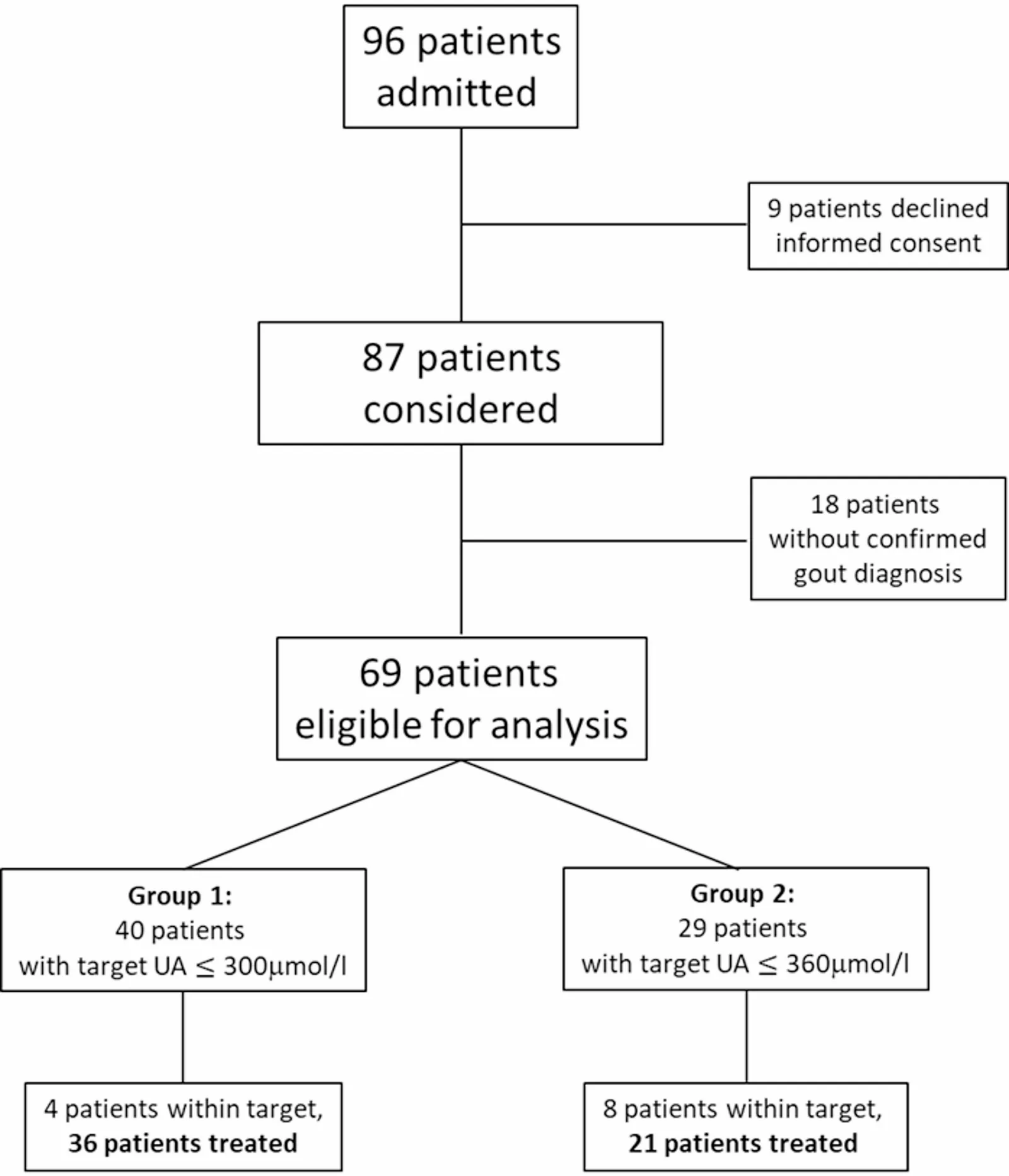
Patient selection tree

A total of 96 patients was admitted (Fig. 1), of which nine patients rejected informed consent for research purposes. For enrollment in the outpatient clinic only patients fulfilling the ACR/EULAR 2015 classification criteria for gout were considered [14]. However, we did not calculate exact scores for classification criteria but only considered patients who fulfilled at least one of the following criteria: positive detection of monosodium urate (MSU) crystals in synovial fluid of a symptomatic joint, positive detection by dual dual-energy CT (DECT), or of a double contour sign (DCS) by ultrasound or classical radiological erosions or clinical detection of tophi. By these means we were unable to confirm gout classification in 18 patients (19%) and these were excluded from the present analysis and not considered for further ULT within our outpatient clinic. The admitting physician was informed about the result of our evaluation.

### Patient grouping

Eventually, 69 patients with confirmed gout were included, of which twelve were already within SU target already at visit 1, resulting in 57 patients, in which *de novo* treatment or treatment escalation was indicated (Fig. 1). Based on the initial assessment patients were divided in two groups defined by their serum-urate (SU) target, according to ACR/EULAR 2016 treatment recommendations [18]: Group 1 (SU target ≤ 300 µmol/L) was defined as severe gout and included patients with tophaceous or erosive gout, or persistent flares despite prior achievement of SU target. Group 2 (SU target ≤ 360 µmol/L) was defined as non-severe gout and included patients with non-tophaceous, non-erosive gout.

Proxy-Comorbidity Indices.

In an attempt to further describe the multimorbidity of our cohort we calculated approximated “proxy-comorbidity scores” for Charlson (CCI [2]), and Elixhauser (applying a van Walraven–like weighting) (ECI [25]), Comorbidity Indices in both groups using available clinical data. For the CCI scoring was as follows: age (+ 1 to + 5 depending on age), diabetes mellitus (+ 1), chronic kidney disease (eGFR < 60 mL/min/1.73 m²; +2). Although transplantation is not part of the original CCI we counted a history of organ transplantation with a score of + 2, as transplantation is usually due to end-stage organ disease and implies lifelong immunosuppression, increased risk of infection, possibly malignancy and cardiovascular complications. The ECI score was approximated using diabetes, chronic kidney disease, arterial hypertension, and transplant status. Scores were calculated consistently across cohorts to allow comparison.

### Intervention

Clinical assessments were performed by an Advanced Practice Nurse (APN) in collaboration with a supervising rheumatologist. The nurse-led gout clinic followed a structured, protocol-based approach and included patient education on disease mechanisms, contributing life-style factors, dietary advice, treat-to-target therapy with urate-lowering medications, and regular monitoring of SU levels and delivery of a standardized information booklet [10]. Pharmacological decisions and prescriptions, including ULT initiation and adjustments, flare prophylaxis (colchicine, prednisone, NSAIDs), and SGLT-2 inhibitor use, were made by rheumatologists according to clinical criteria, updated by 2016 EULAR guidelines [18] and reiterated since then [4].

During the initial ULT titration phase visits were scheduled every 2–4 weeks and included clinical assessment including drug tolerability and, laboratory testing blood count, CRP, SU, creatinine/eGFR, liver enzymes, CK), and documentation of blood pressure, treatment adherence, and flares was performed. Adaptations to ULT and other pharmacological interventions was made upon each visit based on SU as well as drug tolerability.

Allopurinol was the primary ULT, titrated in 50 mg increments for eGFR ≤ 30 mL/min/1.73 m² and 100 mg increments for eGFR ≥ 30 mL/min/1.73 m² until SU targets were reached. Likewise, Febuxostat was used in cases of intolerance to prior allopurinol therapy, starting at 40–80 mg and increased up to 120 mg if needed. Flare prophylaxis followed renal function-adjusted dosing: colchicine 0.5 mg twice daily if eGFR ≥ 30 ml/min/1.73 m² or once daily if < 30 ml/min/1.73 m², or alternatively low-dose prednisone (5 mg daily) or NSAIDs.

Once SU target was reached, flare prophylaxis was stopped (unless there were additional indications, e.g. low-dose prednisone in transplant patients or continuing flares despite SU target). Upon SU target, the referring physician, the general physician and the patient were provided with a detailed written report regarding diagnosis, disease grade (severe, non-severe), SU target level, treatment goal, non-pharmacological and pharmacological intervention and planned follow-up visits at six and twelve months after reaching SU target and with no further appointments scheduled. Patients received information to support self-management of their condition. Specifically, in cases of recurrent flares, loss of urate control, or therapy-related issues, re-referral to the gout clinic was recommended. Patients and physicians were advised to contact us, in case they intended to change ULT dosages. Apart from these recommendations no additional recommendations to external physicians were made.

### Follow-up visits at six and twelve months

Follow-up visits were scheduled at six and twelve months after initially reaching SU target in order to assess maintenance of SU target, treatment adherence, drug tolerability, life style changes and disease activity. Flares were recorded when fulfilling typical inflammatory patterns [16], such as acute pain, swelling, and allodynia, not explained by trauma or exercise. If a patient exceeded his/her SU target, re-initiation (e.g. if treatment was completely stopped since SU target achievement) or adaptations (in case of suboptimal treatment) of ULT were made and follow-up visits were re-scheduled every 2–4 weeks until SU target was reached again and follow-up at six and twelve months re-scheduled thereafter. These cases were counted as additional treatment regimen for analysis purposes. This resulted in higher numbers of treatment regimens than individual patients in some analyses and was specified in the figures and tables, accordingly.

### Assessment of primary and secondary endpoints

The Primary endpoint was % of patients reaching SU target levels in severe a non-severe cohort, respectively. Secondary endpoints included SU median levels, numbers of visits needed to reach SU target, flare rate per group and patient, maintenance of SU target after six and twelve months per group.

### Data analysis

Continuous variables are presented as mean ± SD, categorical variables as percentages. Group comparisons were performed using Welch t-tests or Fisher’s exact test. For comparisons of proxy-CCI and proxy-ECI values Mann Whitney U test was used. A p-value ≤ 0.05 was considered statistically significant.

## Results

### Patient characteristics

Baseline characteristics were comparable between groups (Table 1). Gout was confirmed by MSU, DECT, or DCS in 94% of cases; four patients were clinically diagnosed based on tophi (*n* = 3) or typical MTP I erosions (*n* = 1). Fifteen patients (22%) were transplanted (14 received immunosuppressants, one undergoing CAR-T therapy) highlightening the complexity of this tertiary center gout cohort.

**Table 1:** Baseline parameters of admitted patients with confirmed gout

Parameter	Group 1 (≤ 300µmol/l) Mean (SD) *n* = 40 patients	Group 2 (≤ 360µmol/l) Mean (SD) *n* = 29 patients	*p*-Wert (Welch t-test, *Fisher test, ** Mann-Whitney)
Male %	87%	86%	ns*
Age (years, mean (SD))	64.9 (15.6)	61.6 (20.5)	ns
SU Visit 1 (µmol/l, mean (SD))	449.9 (132.3)	464.6 (123.0)	ns
Gout diagnosed by:			
- MSU in SF - DECT - DCS - Combination of above - clinical (tophy/erosions)	7 15 8 6 4	13 5 7 4 0	N/A
eGFR (ml/min/1.73qm, mean (SD)):	54.3(24.5)	53.2(24.6)	
- proportion with CKD 3 - proportion with CKD 4	35% 20%	34% 28%	ns
CRP mean (mg/l (SD))	17.7 (37)	21.2 (32.1)	ns
BMI (kg/m², mean (SD))	29.7 (6.6)	28.8 (5.8)	ns
Transplanted (n, (%)), treated with: - Tacrolimus - Ciclosporin - Azathioprin (comb) - Mycophenolat (comb) - CAR-T therapy	9 (23%) 4 4 1 1 1	6 (21%) 5 1 0 0 0	ns*
Proxy-Comorbidity Indices:			
- CCI mean, median	5.4, 6	4.8, 5	p_~_0.04**
- Low CCI (0–2) - Medium CCI (3–5) - High CCI (≥ 6)	5% 40% 55%	10% 55% 35%	
- ECI mean	8.5	7.1	p _~_ 0.01**

ns = not significant; SD = Standard deviation; N/A = not applicable; CCI = Charlson Comorbidity Index, incl. proportions of patients with low, medium and high CCI; ECI = Elixhauser Comorbidity Index; CAR-T Chimeric Antigen Receptor T cell; comb = combination therapy; CKD = chronic kidney disease

The age-adjusted Proxy-CCI was higher in patients with severe gout compared to those with non-severe disease (mean CCI 5.6 vs. 4.8). This difference was statistically significant (Mann–Whitney U test, *p* ≈ 0.04). Similarly, the Proxy-ECI comorbidity score was significantly higher in the severe gout cohort (mean 8.5 vs. 7.1; *p* ≈ 0.01).

When stratified by comorbidity burden, patients with severe gout more frequently exhibited high comorbidity (CCI ≥ 6) compared to those with non-severe disease (55% vs. 35%). In contrast, intermediate comorbidity (CCI 3–5) was more common in non-severe gout (55% vs. 40%), while the proportion of patients with low comorbidity (CCI ≤ 2) was low in both groups (10% vs. 5%).

Despite statistically significant differences, substantial overlap in comorbidity distribution was observed between groups, indicating that comorbidity burden alone does not fully explain disease severity in gout.

### Treatment effectiveness

**Fig. 2:**
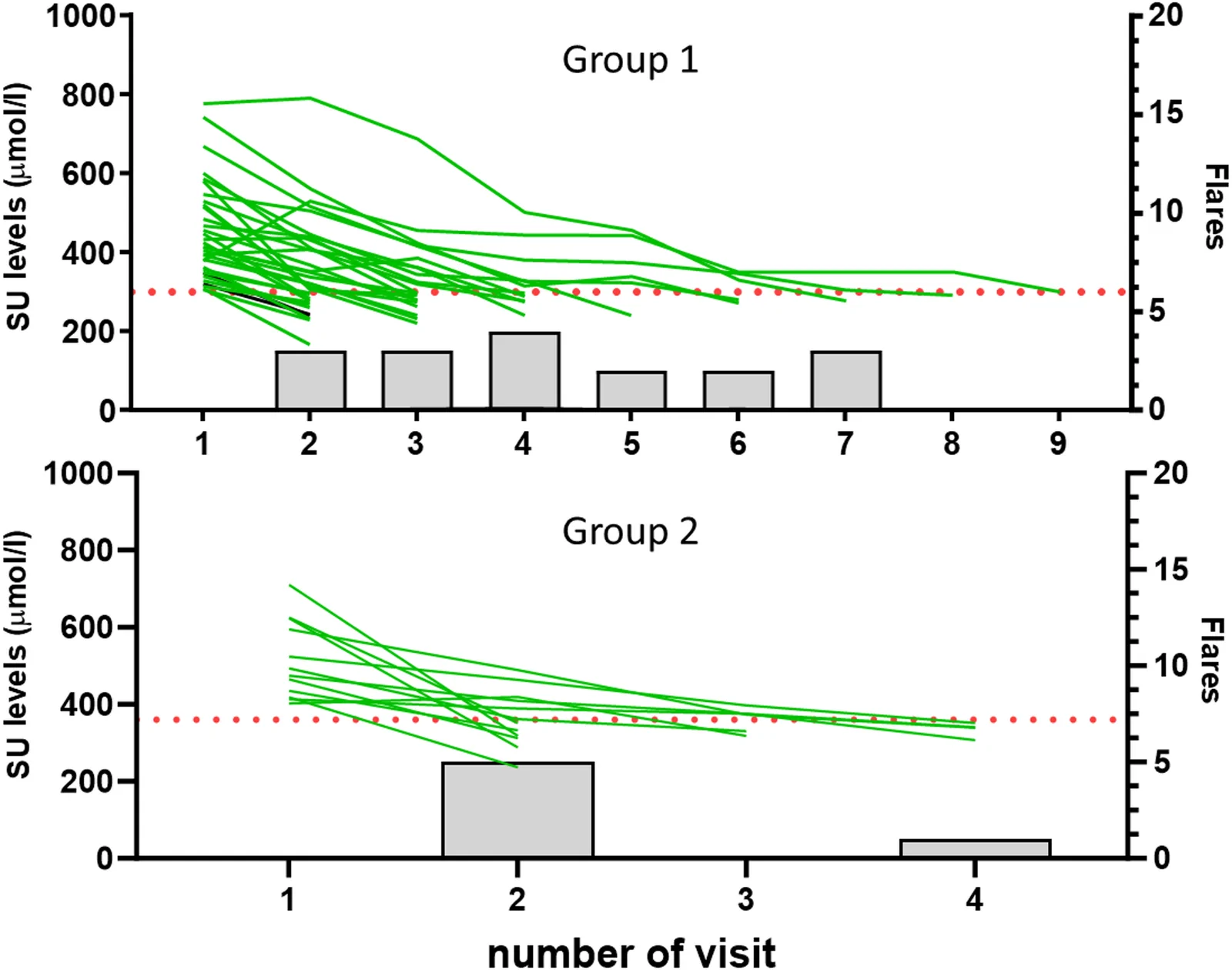
Prospective analysis of SU levels (left y-axis, green lines) and flare frequencies (right y-axis, bar charts) per visit among regimens who successfully reached SU target (dotted red line) in group 1 and group 2. One patient died after reaching SU target (black line, group 1)

In group 1 (*n* = 40), four patients were within SU target at baseline, resulting in 36 treated patients with treatment indication. Considering nine re-initiations/adaptations in this group a total of 45 treatment regimens were implemented. At the time point of the manuscript SU ≤ 300 µmol/L was achieved in 36/45 regimens (80%), with four patients still undergoing ULT titration and considering five drop-outs (Table 2). In group 2 (*n* = 29), eight patients were within SU target at baseline, and 22 regimens (one treatment adaptation) were conducted among the remaining 21 patients. SU target ≤ 360 µmol/L was achieved in 15/22 regimens (68%) at the timepoint of the manuscript, with two patients still undergoing ULT titration and five drop-outs at the timepoint of analysis. Table 2; Fig. 2 show details of and the development of SU levels and flare frequencies during ULT titration for successful regimens. Six patients in group 1 and five in group 2 were excluded from the analysis due to death (*n* = 3, respectively), intolerance to therapy (*n* = 1, respectively) or loss to follow-up (*n* = 2 in group 1, *n* = 1 in group 2) (Table 2).

Group 1 required significantly higher allopurinol doses than group 2 (*p* ≤ 0.01), reflecting lower SU targets and higher prevalence of high-grade renal disease eGFR ≤ 30 ml/min/1.73sqm and, thus, more patients requiring 50 mg increments (*n* = 9 vs. *n* = 5). Flare prophylaxis (colchicine or prednisone) and flare rates were comparable between groups, though one patient in group 1 accounted for 7/17 flares.

**Table 2:** Characteristics of treatment regimens achieving SU target

Variable	Group 1 (≤ 300µmol/l) (*n* = 36 regimens in 27 patients)	Group 2 (≤ 360µmol/l) (*n* = 15 regimens in 14 patients)	Significance (Welch t-test, *Fisher test)
**Treatment period:**			
SU mean at visit 1 (µmol/l, (SD))	441 (124)	496 (101)	ns
with allopurinol	33 (92%)	11 (80%)	ns*
with febuxostat	3 (8.3%)	3 (20%)	ns*
Colchicine prophylaxis	22 (61.1%)	8 (53.3%)	ns*
Prednisolone prophylaxis	10 (27.8%)	4 (26.7)	ns*
NSAR prophylaxis	1 (2.8%)	0	ns*
SGLT2 inhibitors	10 (27.8%)	1 (6.7%)	ns*
**Upon SU target achievement**:			
Total number of visits per group	122	41	ns
Mean (median) visits per patient	3.4 (3.0)	2.7 (2.0)	ns
Mean Allopurinol dose on target (mg/d, (SD))	344 (120)	179 (84)	*p* ≤ 0.01
Mean Febuxostat dose on target (mg/d, (SD)) (*n* = 3 per group)	80 (40)	53.3 (23.1)	N/A
Flares (rate per patient), median	17 (0.47), 0	7 (0.47), 0	ns
**Excluded from analysis (drop-outs):**			
- death - intolerance to treatment - loss to follow-up	3 1 2	3 1 1	

ns = not significant; N/A = not applicable; SD = Standard deviation

### Maintenance of SU-target

At the timepoint of the manuscript a total of 42 treatment regimens (29 in group 1 and 13 in group 2) were followed-up six months after reaching SU target. In addition, 17 regimens (nine in group 1 and eight in group 2) were followed-up twelve months after reaching SU target (Table 3). In group 1, 18/29 (62%) were still within SU-target, a rate which remained largely unchanged after twelve months (*n* = 6/9, 67%). However, in group 2 rates within SU-target were markedly higher at six (*n* = 11/13, 85%) and twelve (*n* = 7/8, 88%) months follow-up, respectively. Of note, most SU exceedances in group 1 were modest (20–30% above target), indicating continued but suboptimal treatment. Among the reasons leading to off-target SU levels we found wrong dosage provided by the nursing home, switch from febuxostat to allopurinol, dose reduction or complete ULT stop by external physicians (external factors), run out of medicine or intake other than prescribed (maladherence) or no clear reason (other). However, in 7/17 (41%) of cases, SU target failures were due to treatment adjustments by external physicians, including complete stop of ULT, with no imminent adverse events attributable to ULT or prior consultation with us.

**Table 3:** Maintenance of SU target levels at six and twelve months follow-up and presumed reasons for SU target deviation, numbers on/off-target refer to treatment regimens

Follow-up (*n*=regimens, patients)	Group 1		Group 2	
	6 months (*n* = 29, 19)	12 months (*n* = 9, 8)	6 months (*n* = 13, 12)	12 months (*n* = 8, 8)
**On target**	8 (62%)	6 (67%)	85% (*n* = 11)	88% (*n* = 7)
**Off-target**				
- external factors - maldherence - other	2 (7%) 6 (21%) 3 (10%)	3 (33%) 0% 0%	2 (15%) 0% 0%	0% 0% 1 (12%)

## Discussion

Our study demonstrates that a supervised nurse-led gout clinic is effective in achieving SU targets in a complex tertiary cohort. 58% of patients had severe gout, and 15 were transplanted, fulfilling at least one criterion of the previously described “difficult-to-treat gout” [15, 20]. In fact, patients with gout have significantly greater odds of accumulating comorbid conditions included in the Charlson comorbidity Index (CCI) (e.g. age, cardiovascular disease, renal impairment, diabetes) compared with matched controls, with adjusted odds ratios increasing with higher CCI categories (e.g., ORs for CCI scores of 1–2, 3–4 and ≥ 5: 1.39, 1.89, and 2.51 respectively) and a shorter time to first comorbidity after diagnosis than controls (median 43 vs. 111 months [11]. In our study both groups 1 and 2 cohorts showed a substantial burden of comorbidity as indicated by adapted comorbidity indices (Proxy-CCI and -ECI). In fact, our values e.g. for proxy-CCI (4.9–5.6) were substantially higher than those of previously reported gout cohorts under primary care (CCI 1.6 [23], or US veterans with confirmed gout (Deyo-CCI 3.1 [22] and even showed CCI ≥ 6 in approximately 45% of patients. In this context, it should be acknowledged that we included only patients who met a restricted subset of the ACR/EULAR 2015 classification criteria for gout, specifically the presence of MSU crystal deposition or pathognomonic clinical signs of gout. Therefore, the study design itself may have introduced a bias toward more severe cases.

Nevertheless, SU targets were typically reached within 2–4 (median 2–3) visits, with allopurinol doses of 200–400 mg in most patients. These findings align with prior primary care studies [8], though our cohort used smaller allopurinol doses (mean 185–347 mg/day vs. 460 mg/day), possibly due to smaller increments for low eGFR (50 mg increments in those with eGFR < 30 ml/min/1.73sqm). Consistently, severe gout patients required significantly higher allopurinol doses and had slower SU reduction (Fig. 2).

Maintenance at six and twelve months was ~ 70% overall, with severe gout patients being less likely to remain at SU target. Interestingly, failure of maintaining SU target levels was due to dose adaptations or even treatment discontinuation by external physicians in almost half of patients. This confirms previous findings from the UK [8] and indicates that both education of patients and attending physicians as well as other health care providers remains an important area to improve interdisciplinary gout management. This may be especially important in multimorbid patients, where more health care providers and an elevated number of communication interfaces exist. Consistently, our findings also suggest that follow-up intervals of six months may be too wide in patients with severe gout in order to maintain SU target levels. New approaches, such as telemedicine [16] and SU self-measurement devices may facilitate SU monitoring [13] and thereby contribute to treatment continuity and better long-term results.

No major ULT adverse effects were observed. Specifically, one patient switched from allopurinol to febuxostat due to intolerance. We observed a mortality rate of 11.1% (6/54 treated patients, three in each group) during the 2.5 year observation period. It Is well known that gout patient show an elevated cardiovascular mortality [3] and based on standardized mortality ratios in gout patients [19], an expected mortality rate of 7–8% can be expected during a 2.5 year interval. In a Danish study on nurse-led gout patients revealed a mortality rate of 15% during 2 years [17]. We therefore consider the observed mortality rate within the expected range.

In summary, our data support the feasibility and effectiveness of nurse-led gout management, with outcomes comparable to those reported previously. However, the complexity observed in tertiary-care gout cohorts appears to be driven primarily by multimorbidity and treatment constraints, suggesting a state of difficult-to-manage gout rather than true difficult-to-treat disease, the latter of which would imply intrinsic therapeutic resistance.

Our study has limitations, namely being a single-center observational study without a control arm and showing a highly biased and possibly heterogeneous gout population, limiting generalizability and causal inference. Second, the sample size was modest, increasing susceptibility to individual patient effects. Thirdly, despite following updated EULAR 2016 treatment guidelines, external provider interventions may have affected SU target maintenance. Lastly, the twelve months follow-up period was rather short and included a low number of patients limiting conclusions on larger and longer scale. On a positive note, however, we herein report on a real-world population, without study biases and can widely reproduce the positive findings of larger studies, despite these limitations.

However, although our results are encouraging, larger multicenter studies are warranted to validate findings and to optimize care including long term adherence in complex gout populations.

## Data Availability

All analyzed data supporting the findings of this study are available within the paper and its Supplementary Information. Raw data of each patient are available upon request.
